# Identification and evolution analysis of *YUCCA* genes of *Medicago sativa* and *Medicago truncatula* and their expression profiles under abiotic stress

**DOI:** 10.3389/fpls.2023.1268027

**Published:** 2023-08-28

**Authors:** An Shao, Shugao Fan, Xiao Xu, Wei Wang, Jinmin Fu

**Affiliations:** Coastal Salinity Tolerant Grass Engineering and Technology Research Center, Ludong University, Yantai, Shandong, China

**Keywords:** *Medicago*, YUC, evolution analysis, expression profile, abiotic stress response

## Abstract

The YUCCAs (YUC) are functionally identified flavin-containing monooxidases (FMOs) in plants that act as an important rate-limiting enzyme functioning in the auxin synthesis IPA (indole-3-pyruvic acid) pathway. In this study, 12 MsYUCs and 15 MtYUCs containing characteristic conserved motifs were identified in *M. sativa* (*Medicago sativa* L.) and *M. truncatula* (*Medicago truncatula* Gaertn.), respectively. Phylogenetic analysis revealed that YUC proteins underwent an evolutionary divergence. Both tandem and segmental duplication events were presented in *MsYUC* and *MtYUC* genes. Comparative syntenic maps of *M. sativa* with *M. truncatula*, *Arabidopsis* (*Arabidopsis thaliana*), or rice (*Oryza sativa* L.) were constructed to illustrate the evolution relationship of the YUC gene family. A large number of cis-acting elements related to stress response and hormone regulation were revealed in the promoter sequences of *MsYUCs*. Expression analysis showed that *MsYUCs* had a tissue-specific, genotype-differential expression and a differential abiotic stress response pattern based on transcriptome data analysis of *M. sativa* online. In addition, RT-qPCR confirmed that salt stress significantly induced the expression of *MsYUC1/MsYUC10* but significantly inhibited *MsYUC2/MsYUC3* expression and the expression of *MsYUC10/MsYUC11/MsYUC12* was significantly induced by cold treatment. These results could provide valuable information for functional analysis of *YUC* genes via gene engineering of the auxin synthetic IPA pathway in *Medicago*.

## 1 Introduction

Auxin is a critical plant hormone, involved in diverse developmental events such as cell division, cell differentiation, and flower development. Indole-3-acetic acid (IAA) is the best-studied naturally occurring active auxin, which are synthesized by two pathways: tryptophan-dependent pathway and tryptophan-independent pathway (Zhao, 2010). For tryptophan-dependent IAA synthesis, there are four proposed branches: (1) indole-3-pyruvic acid (IPA); (2) tyramine pathway; (3) index-3-acetamide pathway; and (4) index-3-acetoxime pathway (Stepanova et al., 2011). Among the four branches, the IPA branch is the major route of IAA biosynthesis inferred by the pleiotropic abnormal phenotype of *Arabidopsis* mutants (Mashiguchi et al., 2011; Won et al., 2011). In the initial step, IPA is catalyzed by Trp aminotransferase 1 (TAA1) and its related proteins TAR1 and TAR2 with Trp as the precursor. Subsequently, the YUCCA (YUC)-encoded enzyme catalyzes the generation of IAA by IPA (Fraaije et al., 2002; Won et al., 2011). The YUC enzyme is the first functionally identified flavin-containing monooxidase (FMOs) in plants. The conserved domain of FMOs contains two conserved motifs, the flavin purine dinucleotide (FAD) binding site and the reduced coenzyme binding site (NADPH-binding site), which have the same G_X_GxxG characteristic structure in their amino acid sequences (Zhao, 2012).

The *YUC* gene was originally identified from *Arabidopsis* mutants with reduced IAA content (Zhao et al., 2001). Genetic and physiological analyses of the loss-of-function mutants of the *YUC* gene have further demonstrated its important role and rate-limiting enzyme function in the auxin synthesis IPA pathway. Overexpression of transgenic *Arabidopsis* lines of the *YUC* gene showed slightly increased auxin levels, accompanied by phenotypic including hypocotyl elongation, cotyledon bias, and enhanced apical dominance (Zhao et al., 2001). Subsequent studies showed that overexpression of the *YUC* gene in plants such as rice, potato, and strawberry could also produce similar phenotypes of auxin overproduction (Kim et al., 2012; Liu et al., 2014). In addition, inactivation of a single *YUC* gene in *Arabidopsis* presented not obvious developmental defects, whereas multiple mutants plants have more severe phenotypes (Cheng et al., 2006), suggesting functional redundancy among *YUC* members. Moreover, gene and protein expression data in *Arabidopsis* indicated that *YUC1*, *2*, *4*, and *6* were mainly expressed in the stems, whereas *YUC 3*, *5*, *7*, *8*, and *9* were mainly functional in the roots (Chen et al., 2014). The *yuc1yuc2yuc4yuc6* quadruple mutants had severe defects in vascular patterning and failed to produce a normal inflorescence but had no root defects, consistent with their stem-localized expression pattern. *YUC3*, *5*, *7*, *8*, and *9* are expressed during root development, and the multiple mutants of the five *YUC* genes developed short and agrotropic roots (Chen et al., 2014). In addition, *YUC* genes expressed in the shoots (*YUC 1*, *2*, *4.1*, and *6*) are localized to the cytoplasm, whereas root *YUC* genes are the ER (endoplasmic reticulum) membrane-binding proteins. In addition, the phenotypes of different sets of individual YUC knockout mutants cannot be complemented by the expression of *YUC* genes expressed in other tissues (Chen et al., 2014; Zhao, 2018). These studies suggested that different sets of *YUC* genes exhibited tissue expression specificity, organ-specific subcellular localization patterns, and differential of gene function for auxin biosynthesis.

Plants often respond to environmental stress by regulating hormonal pathways. Several studies have shown that the auxin biosynthetic pathway is upregulated in response to certain abiotic stresses including regulating the expression of *YUC* genes (Blakeslee et al., 2019). For example, several root-specific *YUC* genes have been reported to mediate aluminum stress-induced inhibition of root growth in *Arabidopsis* (Liu et al., 2016). Heat and low-temperature stress can induce ER sheet formation by inducing a specific *YUC* gene (Pain et al., 2019). In *Arabidopsis*, heat stress led to an indirect increased expression of *YUC8* (Sun et al., 2012), which is similar to the upregulation of *CsYUC8/9* in cucumber. Cold stress also led to the upregulation of CsYUC10b but downregulation of other CsYUC proteins in cucumber (Yan et al., 2016). RNA-seq analysis of *Arabidopsis* under heat and drought stress also revealed a tissue-specific difference in the up- or downregulation of *TAA/YUC* auxin biosynthesis genes, such as the upregulation of *YUC9* expression in leaf tissues after heat stress (Blakeslee et al., 2019). Overexpression of *YUC7* in *Arabidopsis* (Lee et al., 2012), and *YUC6* in potato was able to increase drought tolerance with reduced water loss in transgenic plants by reducing the decomposition of IAA (Kim et al., 2012; Cha et al., 2015). An increased free IAA level and improved drought stress tolerance connected with reduced levels of reactive oxygen species and delayed leaf senescence have been observed for plants such as tomato, maize, rice, and petunia (Ke et al., 2015). In contrast to most results in *Arabidopsis*, increased drought tolerance associated with decreased root IAA levels in rice was found, accompanied by the downregulation of various *YUC* genes (Du et al., 2013; Naser and Shani, 2016). The different expression patterns of *YUC* genes in response to different stresses or in different species suggested a possible functional differentiation of *YUC* genes during stress response.

*Medicago sativa* L. is a perennial herbaceous legume forages with high yield, nutrient value, and palatability. As a basic component in rations for animals and an important cash crop for biofuel ethanol production, it is widely cultivated (Li et al., 2011). However, the growth and yield of *M. sativa* could be severely inhibited by external stresses such as salt, cold, and drought stress. Recently, large-scale potential genes involved in *M. sativa* responsive to adverse stimuli have been investigated by transcriptional profiling and detected several stress-responsive genes and categories (Postnikova et al., 2013; An et al., 2016; Luo et al., 2019; Ma et al., 2021). Root and leaf transcriptomes under salt stress revealed a hormone interaction involved in salinity adaptation (Lei et al., 2018). Overexpressing IAA within root nodules of *M. sativa* was associated with the improved drought tolerance of plants (Defez et al., 2017). Although *YUCs* have been identified in several species of plants, such as 11 *AtYUCs* in *Arabidopsis* (Mashiguchi et al., 2011), 7 *OsYUCs* in rice (Yamamoto et al., 2007), 22 *TaYUCs* in wheat (Yang et al., 2012), 22 *GmYUCs* in soybean (Wang et al., 2017) and 14 *ZmYUCs* in maize (Li et al., 2015), the IAA biosynthesis-related *YUC* genes in *M. sativa* or its model legume species *M. truncatula* (*Medicago truncatula* Gaertn.) has not yet been identified at the genome-wide level and the tissue-specific and abiotic stress expression patterns have not been analyzed (Li and Brummer, 2012), greatly limiting the improvement of stress adaptability of *M. sativa* by modifying the auxin pathway through genetic engineering.

In this study, a total of 12 *MsYUCs* in *M. sativa* and 15 *MtYUCs* in *M. truncatula* were identified. The gene structure, motif composition, chromosome location, and gene replication events were analyzed, and the evolutionary relationship of other species associated with *M. sativa* was constructed. An overall comparative expression analysis in *M. sativa* was performed to examine the *YUC* gene expression patterns in different tissues, different varieties, and their responses to cold, drought, and salt stress. These results could provide valuable information for identifying candidate *MsYUCs* involved in different biological processes and various abiotic stress responses in *M. sativa* for further gene functional study and for genetic modification.

## 2 Materials and methods

### 2.1 Identification of *Medicago YUC* genes and basic characteristic analysis

Genome sequence and genome annotation information of *M. sativa* variety Zhongmu No. 1 and *M. truncatula* used in this study were downloaded from Ensembl Plants (https://plants.ensembl.org). The amino acid sequences of the *Arabidopsis* YUC family members were downloaded from the TAIR website (https://www.arabidopsis.org/) and used as Query to search the *Medicago* protein sequences by Local BLAST, and the sequences with e-value less than −20 were reserved. The latest version of all schema database files “Pfam-a.hm.gz” from the Pfam database (https://pfam.xfam.org/) were downloaded, and candidate YUC members containing the FMO-like domain (PFam00743) were identified using TBtools’ (Chen et al., 2020) simple HMM Search plug-in. Results obtained from BLAST and Pfam search were further merged to remove duplicates. Finally, the Batch CD search function in the NCBI website (https://www.ncbi.nlm.nih.gov/) and the SMART database were used to detect and retain the correct and complete sequences of YUC characteristic conserved motifs (http://smart.embl-heidelberg.de/). The basic features such as molecular weight were determined, and isoelectric point analysis was performed using the ExPASy Proteomic Server (https://web.expasy.org/protparam/).

### 2.2 Chromosome localization and conserved motif and gene structure analyses

According to the chromosomal location data contained in the downloaded *Medicago* genome annotation information, TBtools was used to map the chromosomal location of YUC members. The YUC members detected in *Medicago* were named according to their position from top to bottom on chromosomes 1–8. The conserved motif of *YUC* genes was identified using online motif detection software (http://meme.nbcr.net/meme/), and the length of the motif was set from 2 to 200 bp to detect a maximum of 12 motifs. Visualization was performed with the TBtools software. For gene structure analysis, TBtools’ “gene structure view” function was used to visualize the gene structure (exon and intron number and location) of MsYUC family genes. The “One step build ML tree” plug-in of TBtools was used to get a Newick tree and displayed in the front of the conversed motif and gene structure exhibition.

### 2.3 Phylogenetic and gene duplication and synteny analysis

A phylogenetic tree was constructed using the YUC amino acid sequences of *Arabidopsis*, rice, *M. truncatula*, and *M. sativa* to analyze the homology relationships. All YUC sequences were aligned to multiple sequences using Clustal W, and the alignment resulted in phylogenetic tree construction using MEGA6.0 software (Larkin et al., 2007; Tamura et al., 2013). The establishment method used the adjacency method (neighbor-joining method) and the *P*-distance model with the bootstrap test for 1,000 times. Replication events of *Medicago YUC* genes and collinear blocks of *YUC* genes within *M. sativa*, *Arabidopsis*, *M. truncatula*, and rice were analyzed using the “One Step MCScanX Wrapper” function of TBtools with the e-value of 1e^−3^ and number of blast hits of 10. Tandem and segmental duplicates in the YUC gene family were identified using TBtools by searching the final “tandem” and “gene Linked Region” files after running. Phylogenetic analysis of species was performed using “phyliptree.phy” derived from the “NCBI Taxonomy” function. The Ka (nonsynonymous) and Ks (synonymous) substitution rates of gene duplication pairs were calculated using the “Simple Ka/Ks Calculator” function of TBtools. Ka/Ks <1, = 1, and >1 represent purification selection, neutral selection, and positive selection, respectively (Zhang et al., 2006). The divergence time (million years ago/MYA) was calculated through formula T = Ks/2λ * 10^−6^ (λ = 6.5 × 10^−9^).

### 2.4 Protein structure and subcellular localization prediction

Secondary structure prediction of MsYUCs was performed by Phyre2 (http://www.sbg.bio.ic.ac.uk/servers/phyre2/html/page.cgi?id=index). A tertiary structure model of the MsYUC proteins was predicted by SWISS-MODEL (https://swissmodel.expasy.org//). Global model quality estimation (GMQE) was used to obtain the high score-predicted model. Trans-membrane domain (TMD) prediction was constructed using TMHMM based on the hidden Markov model (https://services.healthtech.dtu.dk/services/TMHMM-2.0/). Using the online website CELLOv.2.5, subcellular localization was predicted (http://cello.life.nctu.edu.tw/ ) (Yu et al., 2010).

### 2.5 Analysis of the promoter-based cis-acting elements

Promoter sequences of the *MsYUC* genes (2,000 bp upstream of the ATG) were extracted by the “GTF/Gff3 Sequence Extract” function of TBtools using “genome annotation file” and “genome fasta file.” The promoter sequences were submitted to the PlantCARE (http://bioinformatics.psb.ugent.be/webtools/plantcare/html/) website for cis-acting element analysis, and the elements represented by different-colored symbols were visualized using TBtools’ “Basic Biosequence viewer” function.

### 2.6 Analysis of *MsYUC* gene expression patterns in the RNA-seq data

RNA-seq data of different tissue were downloaded from the online LegumeIP V3 website (https://www.zhaolab.org/LegumeIP/gdp). The expression data of different genotypes and under various abiotic stresses were obtained from previous studies (Zhou et al., 2018; Luo et al., 2019). The different expression profiles were exhibited through a heat map constructed by “Amazing Heatmap” function of TBtools.

### 2.7 RT-qPCR analysis

Eight-week-old seedlings of Zhongmu No. 1 were exposed to untreated control (CK), cold (4°C), and salt (200 mM NaCl) stresses for 6 h. After treatment, RNeasy Kit (Qiagen) was used to extract the total RNA from three biological replicates under control, salt, and cold stresses, respectively. First-strand cDNA of each sample was synthesized using the TaqMan reverse transcription kit (Applied Biosystems). qPCR was conducted on an ABI real-time PCR system with a total volume of 20 μl containing 10 μl of SYBR Green real-time PCR master mix (Toyobo, Japan), 2 μl of cDNA template, 0.2 μM of upstream and downstream primers. The qPCR program was conducted with denaturation at 95°C or 10 min, followed by 40 cycles of amplification (95°C for 30 s, 60°C for 30 s, and 68°C for 1 min) using the ABI real-time PCR system (Applied Biosystems, Foster City, CA). Transcript levels of each sample were determined and normalized to the untreated control sample (CK) as a calibrator with respect to the internal control gene using the 2^−ΔΔCt^ method (Schmittgen and Livak, 2008). Values represent mean ± *SD* of three biological replications. One-way ANOVA test was used, and significant differences from CK and treated plants at *P* < 0.05 are shown by asterisks. All the technical aspects of qPCR experiments fitted the MIQE Guidelines (Bustin et al., 2009). The primers used are listed in **Table S1**.

### 2.8 Protein–protein interaction and miRNA target prediction

All MsYUC protein sequences were submitted to the STRING website (http://string-db.org) to build a protein–protein interaction network with their *Arabidopsis* orthologs as a reference. Using *M. truncatula* miRNAs as reference, target miRNAs were predicted through the psRNATarget website (https://www.zhaolab.org/psRNATarget/) with default parameters while selecting target accessibility, as previously described (Dai et al., 2018).

## 3 Results

### 3.1 Identification and basic characterization of the MsYUC and MtYUC gene families

Comparative homology analysis was performed using the downloaded *Arabidopsis* YUC protein sequences as Query to search the protein sequences and the genome sequence of *Medicago*, and a total of 12 *MsYUCs* and 15 *MtYUCs* were identified from *M. sativa* and *M. truncatula*, respectively. All members were designated *MsYUC1-MsYUC12* and *MtYUC1-MtYUC15* according to their distribution and location information on the chromosome (**Table 1**; **Figure S1**). The *MsYUC* genes showed a significant uneven distribution on eight chromosomes, with the most four *MsYUC* genes on chromosome 1, three *MsYUC* genes on chromosomes 3 and 7, and only one *MsYUC* gene on chromosomes 5 and 6, but no distribution of *MsYUC* genes on chromosomes 2, 4, and 8 (**Figure S1A**). However, *MtYUC* genes were distributed in all chromosomes except for chromosome 2 (**Table 1**; **Figure S1B**). Chromosomal localization also showed that all *YUC* genes could be localized to the *Medicago* chromosomal genome. As shown in **Table 1**, the length of the coding region (ORF) of *MsYUC* genes varied from 360 to 573 amino acids with a molecular weight (MW) from 40.97 to 64.11 kD. The ORF and MW of *MtYUC* genes varied in relatively small ranges, with The ORF from 382 to 430 amino acids and MW from 42.87 to 48.28 kD (**Table 1**). All YUC proteins were basic proteins with isoelectric points (pI) greater than 8 (ranging from 8.1 to 9.12). Subcellular location prediction showed that both MsYUC and MtYUC proteins had cytoplasmic and periplasmic locations (**Table 1**).

**Table 1:** Characteristics of the YUC gene family members in *Medicago*.

ID	Name	ORF	Start	End	W/Da	pI	Location
MsG0180001906.01	MsYUC1	423	29138325	29139895	47167.58	9.12	Periplasmic
MsG0180002563.01	MsYUC2	385	40447849	40451427	43510.12	8.67	Cytoplasmic
MsG0180002571.01	MsYUC3	385	40561568	40564952	43526.16	8.78	Cytoplasmic
MsG0180003762.01	MsYUC4	420	67805768	67807662	47025.45	9.01	Cytoplasmic
MsG0380016438.01	MsYUC5	527	83416606	83420598	59400.64	8.98	Periplasmic
MsG0380016439.01	MsYUC6	360	83450071	83452625	40965.77	9.1	Periplasmic
MsG0380017591.01	MsYUC7	425	98226131	98230092	47542.93	8.82	Cytoplasmic
MsG0580025734.01	MsYUC8	511	23344641	23350209	57393.75	8.63	Periplasmic
MsG0680035661.01	MsYUC9	573	110273357	110282037	64111.41	8.78	Cytoplasmic
MsG0780040831.01	MsYUC10	416	83110763	83112388	46856.19	8.62	Cytoplasmic
MsG0780041255.01	MsYUC11	419	88839687	88843914	47701.96	8.7	Cytoplasmic
MsG0780041256.01	MsYUC12	383	88846915	88850153	43072.34	8.1	Periplasmic
AES58795	MtYUC1	430	883915	889499	48280.0	8.87	Cytoplasmic
AES58948	MtYUC2	406	2133963	2136290	45687.8	8.91	Periplasmic
KEH41176	MtYUC3	423	17407546	17409111	47142.53	9.12	Periplasmic
KEH42432	MtYUC4	421	29855829	29858303	47130.5	8.95	Cytoplasmic
KEH35392	MtYUC5	398	40705084	40707031	44902.85	8.38	Periplasmic
KEH35393	MtYUC6	391	40711936	40713932	44194.08	8.73	Periplasmic
KEH35394	MtYUC7	398	40720455	40722348	44929.91	8.38	Cytoplasmic
AES73853	MtYUC8	423	50666906	50671263	47154.53	8.81	Cytoplasmic
KEH29783	MtYUC9	399	18624417	18627401	45407.79	8.99	Cytoplasmic
AES96101	MtYUC10	382	14352361	14354443	42872.11	8.7	Cytoplasmic
KEH27129	MtYUC11	408	32789500	32793211	45302.98	9.1	Cytoplasmic
AES81674	MtYUC12	416	39838750	39840931	46783.18	8.72	Cytoplasmic
KEH24362	MtYUC13	383	43925708	43928430	43201.76	8.95	Cytoplasmic
KEH24364	MtYUC14	384	43937223	43940456	43148.61	8.7	Periplasmic
KEH18954	MtYUC15	382	12379792	12381700	42913.26	8.42	Cytoplasmic

### 3.2 Phylogenetic analysis of *Medicago YUC* proteins

**Figure 1:**
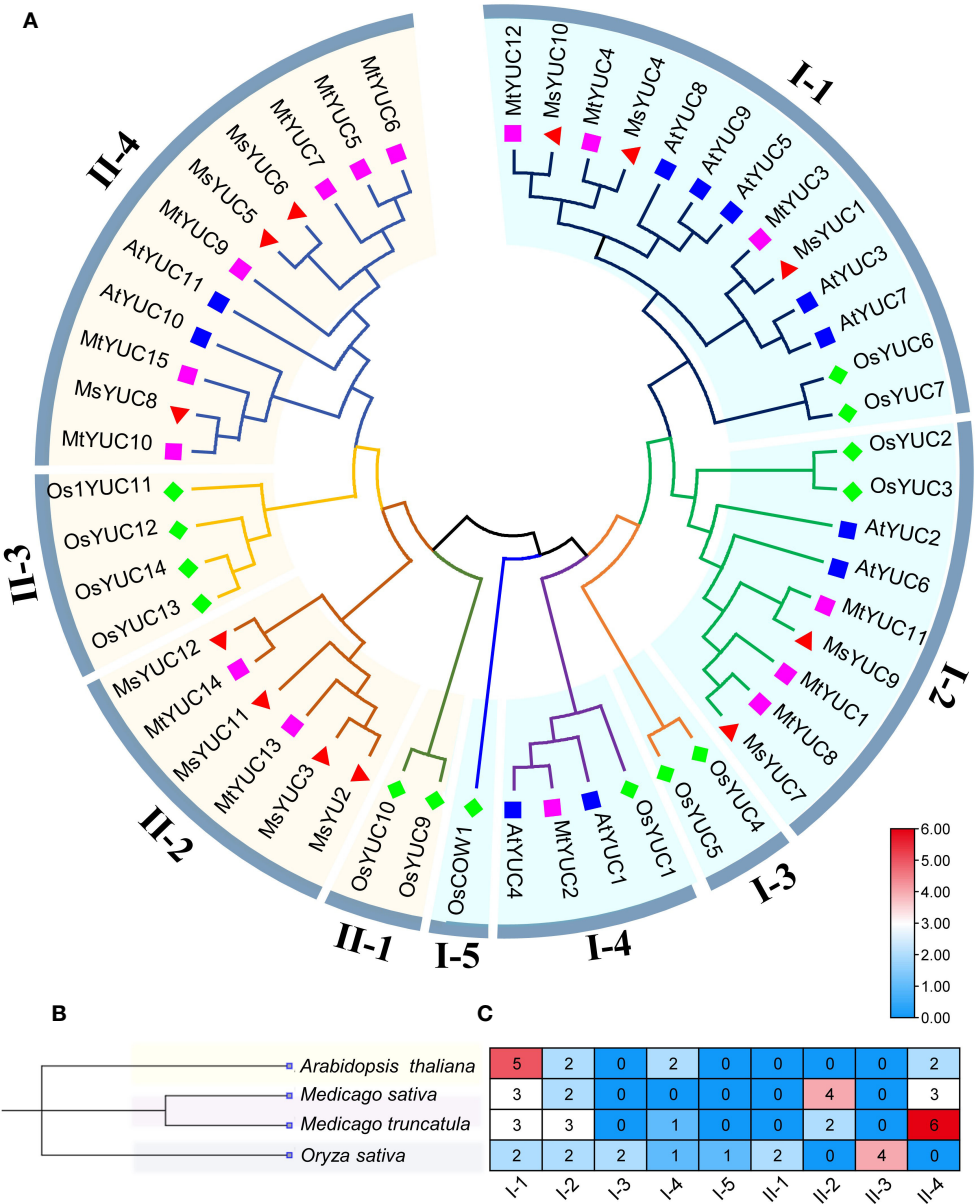
The phylogenetic analysis and subfamily clusters of YUC proteins in plants. **(A)** Phylogenetic analysis using YUC proteins in *Medicago* (MsYUC and MtYUC), *Arabidopsis* (AtYUC), and rice (OsYUC). The phylogenetic tree was constructed using the ClustalX program and the neighbor-joining method. **(B)** Evolutionary relationships among four species. Phylogenetic analyses of four species were performed using “phyliptree.phy” from the NCBI Taxonomy function. **(C)** Number of YUC proteins in four species and their distribution in various subfamilies.

To further analyze the kinship of *YUC* genes, YUC protein sequences from *M. sativa* (12 MsYUC), *Arabidopsis* (11 AtYUC), *M. truncatula* (15 MtYUC), and rice (14 OsYUC) were selected, and an evolutionary tree was constructed (**Figure 1A**). The results showed that 52 YUC proteins in four species can be clustered into two large clusters (clade I and clade II). Clade I can be further subdivided into five small clades, with MsYUC1, 4, 10 and MtYUC3, 4, 12 in clade I-1, MsYUC7, 9 and MtYUC1, 8, 11 in clade I-2. Clade II can be subdivided into four small clades, with MsYUC2, 3, 11, 12 in Clade II-2, MsYUC8, 5 and 6 in clade II-4. MtYUC2 showed a close relationship with AtYUC1 and AtYUC4, which were clustered into clade I-4. MsYUC7/9 and MtYUC1/8/11, belonging to clade I-2, were relatively closely related to AtYUC6. MtYUC10, MtYUC15, and MsYUC8 were closely related to AtYUC10, which belongs to clade II-4 (**Figure 1A**). A phylogenetic tree of four species was constructed, and the number and distribution of YUC proteins in various subfamilies in four species were counted (**Figure 1B**). Notably, clade I-3 and cladeII-3 only contained YUC proteins from rice and clade I-4 only contained YUC proteins from the other three species except *M. sativa*. Clade II-2 only contained YUC members from *Medicago* with no homologous gene from *Arabidopsis*. Clade II-4 contained YUC proteins from the other three species except rice. Only two clades, clade I-1 and clade I-2, both contained YUC proteins from the four species (**Figure 1C**).

### 3.3 Gene duplication, synteny, and evolution analysis of the YUCs

**Figure 2:**
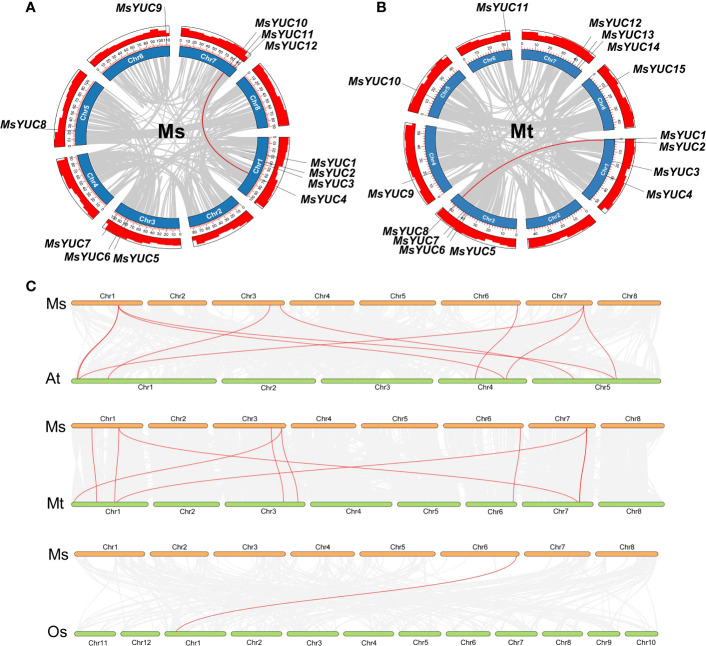
Duplication event analysis for the YUC gene family in the *M. sativa* (Ms) and *M. truncatula* (Mt) genome and synteny analysis between *M. sativa* and the other three species. The duplication events in the *M. sativa* genome **(A)** and *M. truncatula* genome **(B)**. Red-colored lines indicate duplication events of MsYUC family members (*MsYUC10*/*4*) and MtYUC family members (*MsYUC1/8*). **(C)** Collinearity analysis of *M. sativa* (Ms) with *M. truncatula* (Mt) o*r Arabidopsis* (At) or rice (Os). Red-colored lines indicate the YUC family members in different species.

Tandem and segmental duplication events were analyzed to further investigate the evolutionary pattern of the YUC gene family in *Medicago*. Results revealed that *MsYUC5/MsYUC6* on chromosome 3 and *MsYUC11/MsYUC12* on chromosome 7 were obvious tandem duplication genes (**Figure S1A**). Only one *MsYUC* gene pair (*MsYUC4/MsYUC10*) could be identified as segmental duplication events (**Figure 2A**). The MtYUC gene family has an additional tandem repeat gene pair on chromosome 3 (*MtYUC5/6*, *MtYUC6/7*) (**Figure S1B**; **Table S1**). Only one *MtYUC* gene pair (*MtYUC1/MtYUC8*) of *M. truncatula* was identified as segmental duplication genes (**Figure 2B**). Comparative syntenic maps of *M. sativa* with *Arabidopsis*, rice, and *M. truncatula* were constructed to illustrate the evolution relationship of the *YUC* gene family (**Figure 2C**). Notably, 10, 10, and 1 orthologous pairs were found between *M. sativa* and *Arabidopsis*, *M. sativa* and *M. truncatula*, and *M. sativa* and *O. sativa*, respectively (**Figure 2C**). The collinear blocks in which *MsYUC4, 5, 7, 9, 10* were located is present in *M. truncatula* and *Arabidopsis* except rice. *MsYUC1*-related collinear blocks were found only in *Medicago* but not in *Arabidopsis* or rice (**Table S2**). Interestingly, one MsYUC family member, *MsYUC9*, had collinear relationships with gene(s) in all species analyzed (**Table S2**). The Ka/Ks ratio of homologous *MsYUC* gene pairs ranged from 0.19 (*MsYUC4/10*) to 0.49 (*MsYUC5/6*), whereas the Ka/Ks ratio of *MtYUC* homologous ranged from 0.09 (*MtYUC5/6*) to 0.24 (*MtYUC6/7*), indicating that the *YUC* genes of *Medicago* had undergone a great purification selection pressure (**Table S3**). The evolutionary divergence time (MYA) calculated showed that two homologous gene pairs *MsYUC4/10* (47.83 MYA) and *MtYUC1/8* (57.28 MYA) were derived from the formation period of genus *Medicago*. Three gene pairs of *MsYUC5/6*, *MtYUC5/6*, and *MtYUC6/7* homologous gene pairs were derived around 5 million years ago, and one homologous gene pair (*MsYUC11/12*) was derived around 75.77 MYA (**Table S3**).

### 3.4 Motif and gene structure analysis of MsYUC members

**Figure 3:**
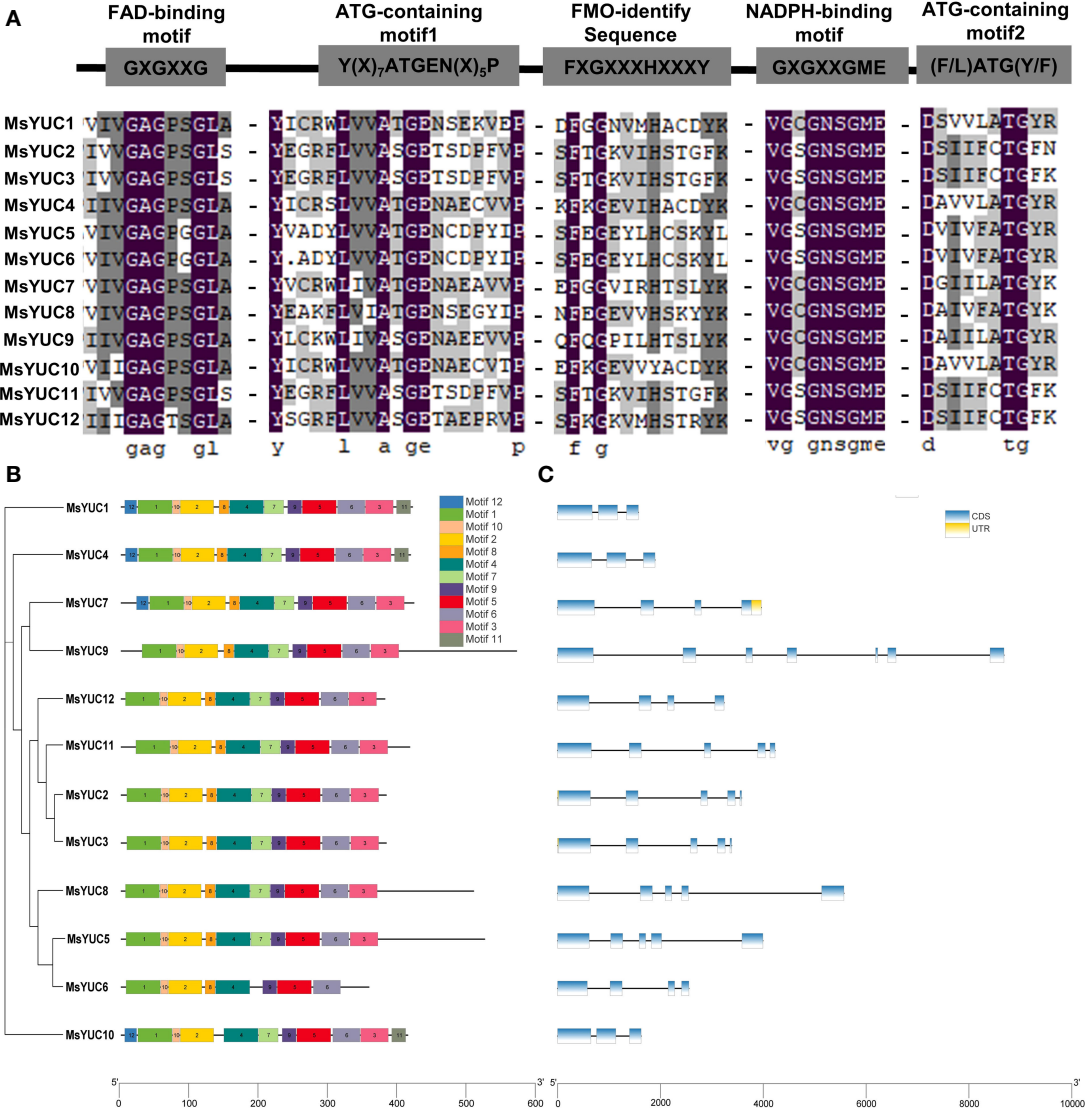
Structure and conversed motifs of MsYUC members. **(A)** Alignment of conserved domains in MsYUC proteins. **(B)** Conserved motifs of *MsYUC* genes predicted by MEME. **(C)** Gene structure of *MsYUC* genes. The exons are represented by blue boxes, and black lines are represented by black lines.

Motif analysis showed that 12 MsYUC proteins all contained the conserved FAD-binding motif and NADPH-binding motif (**Figure 3A**), suggesting a conserved function. Nevertheless, some differences were observed in the FMO-identifying motif of MsYUC5/6/10 proteins, ATG-containing motif1 of MsYUC2/3/9/11/12 proteins, and ATG-containing motif2 in MsYUC2/3/11/12 proteins, which might contribute to the functional divergences (**Figure 3A**). In the prediction analysis of the conserved motif of MsYUC proteins, 12 relatively conserved motifs (motifs 1~12) were further identified, including motif1 as the FAD binding site and motif2 as the reduced NADPH binding site (**Table S4**; **Figure 3B**). Furthermore, eight conserved motifs (motif1, 2, 4, 5, 6, 8, 9, 10) were present in all MsYUCs examined. Each MsYUC protein contained a minimum of 8 to a maximum of 12 of these motifs. and MsYUC6 protein had the least motif. MsYUC1 and MsYUC4 protein had all 12 conserved motifs and MsYUC7 had 11 conserved motifs except motif12. Seven MsYUC proteins (MsYUC9/12/11/2/3/8/5) contained the same 10 conserved motifs (motif1~10). Only MsYUC10 protein lacked motif8 compared with other members (**Figure 3B**). Gene structure analysis revealed that the number of exons of *MsYUCs* varied from 3 to 7 whereas *MsYUC9* contained the most numerous introns. Five *MsYUC* genes (*MsYUC2/3/5/8/11*) had five exons. Three *MsYUC* genes (*MsYUC7/12/6*) had four exons, and three *MsYUC* genes (*1/4/10*) had three exons. Seven *MsYUC* genes (*MsYUC12/11/2/3/8/5/6*) containing the same 10 conserved motifs had four exons, whereas three members had only two exons (*MsYUC1/4/10*) (**Figure 3C**).

### 3.5 Expression analysis of *MsYUCs* in different tissues and different genotypes

**Figure 4:**
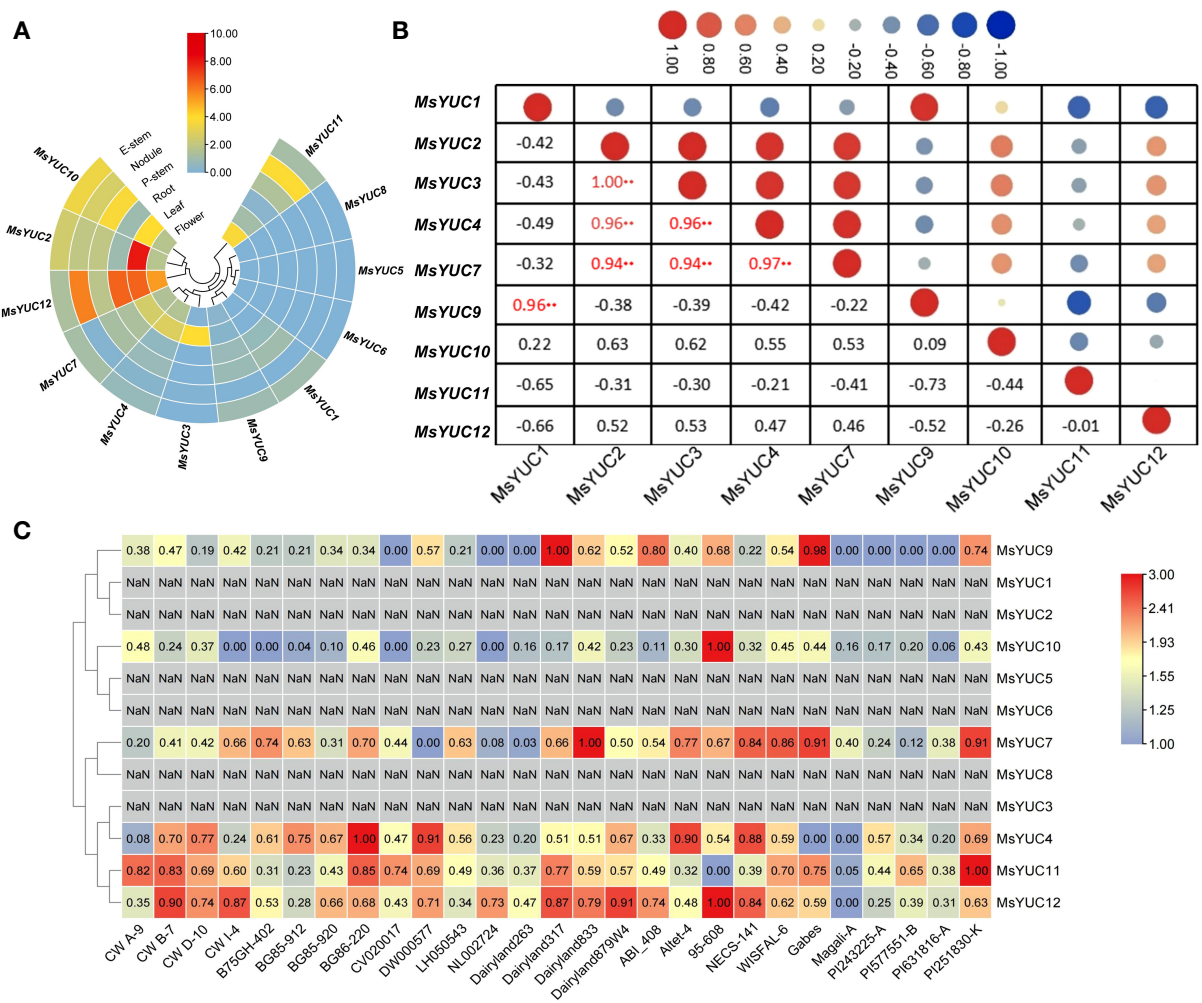
Expression pattern of *MsYUC* genes in different tissues and in different genotype. **(A)** Tissue-specific expression analysis of *MsYUC* genes. **(B)** The correlation of gene expression patterns between every two *MsYUC* genes. Red and blue circles represent positive and negative correlations, respectively. **(C)** *MsYUC* expression in different genotypes. The color scale of the heatmap refers to the relative expression level.

The expression patterns of 12 *MsYUC* genes in different tissues were examined using online transcriptome data. Results indicated a tissue expression specificity of different *MsYUC* genes. For example, *MsYUC10*, *MsYUC12*, and *MsYUC2* had relatively higher expression levels in specific tissues examined, whereas some members (*MsYUC5/6/8*) had very low expression levels and were barely detectable. In addition, *MsYUC2* had a higher expression level in leaves than in other tissues and *MsYUC12* was more highly expressed in both leaves and roots than in other tissues (**Figure 4A**). In addition, we further analyzed the expression correlation between every two *MsYUC* genes in five tissues. *MsYUC2* showed to be significantly positively correlated with *MsYUC3*, consistent with their close relationship in the phylogenic tree. *MsYUC2* and *MsYUC3* showed to be significantly positively correlated with *MsYUC4* and *MsYUC7*, respectively. *MsYUC3* showed to be significantly positively correlated with *MsYUC4* and *MsYUC7*, respectively. *MsYUC4* and *MsYUC7*, as well as *MsYUC1* and *MsYUC9*, were significantly positively correlated (**Figure 4B**). There was also a differential expression pattern of *MsYUCs* among different genotypes. For example, *MsYUC10* and *MsYUC12* showed a higher expression level in 95-608 compared with other genotypes. *MsYUC11* had a relatively higher expression level in PI251830-K but had the lowest expression detected in 95-608 (**Figure 4C**).

### 3.6 Promoter analysis and stress response expression of *MsYUCs*

**Figure 5:**
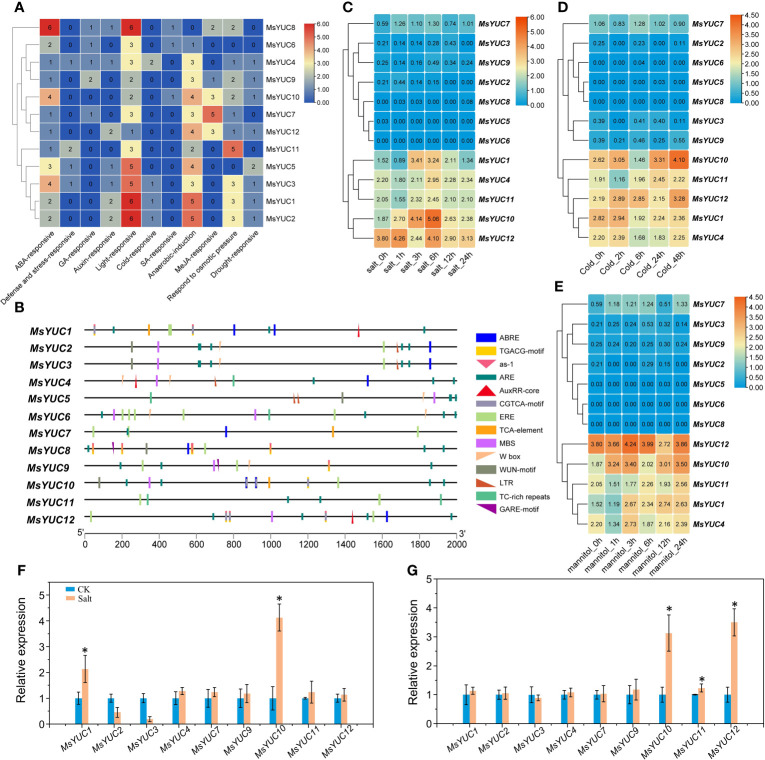
Cis elements of *MsYUC* promoter prediction and expression analysis of *MsYUCs* under stress conditions. **(A)** Number of hormone and stress response-related elements of *MsYUCs*. **(B)** Main elements distributed in the promoter region of *MsYUC* genes. Expression of *MsYUC* genes under salt stress **(C)**, mannitol treatment **(D)**, and cold stress **(E)**. The color scale of the heatmap refers to the relative expression level. Relative expression of *MsYUCs* treated by NaCl **(F)** and cold **(G)** determined by RT-qPCR. Three replicates were designed for each sample, and *M. sativa* actin gene expression was used for data normalization. Value represents mean ± *SD* of three replicates. * indicated significant different from untreated control (CK) plants (p < 0.05, one-way ANOVA).

To predict the possible regulation of *MsYUCs* expression, the cis-acting elements included in the promoter sequence of the *MsYUC* genes were analyzed. Results revealed a variety of stress response elements related to hormone and stress response (**Figure 5A**). Auxin-responsive elements were found in the promoter region of *MsYUC1*, *4, 5, 8, 12*, and three of their promoters contained AuxRR-core elements. The promoter of *MsYUC8* had the most cis-acting elements (6) involved in the abscisic acid responsiveness (ABRE) (**Figure 5B**). The *MsYUC7* promoter region contained five CGTCA motifs, which functions in Me-JA responsive. There were also some GA-responsive elements such as GARE-motif, TATC-box, P-Box, and some SA-responsive elements (TCA-element) in the promoters of certain *MsYUCs* (**Figure 5A**). The promoter of *MsYUC6*, *8*, and *10* contained one cis-acting element involved in low-temperature responsiveness LTR (CCGAAA), respectively. Except for *MsYUC6*, *7*, and *8*, other members all had one or two MYB-binding site (MBS) involved in drought inducibility. Some anaerobic induction, osmotic pressure-responsive, and defense and stress-responsive elements (TC-rich repeats) were also present on certain *MsYUC* promoters (**Figure 5A**). In addition, all the *MsYUC* promoters had light-response elements. *MsYUC1, 2, 8* had the most light-response elements (6) whereas *MsYUC12* had the least light-response element (1) (**Figure 5B**). Based on the abiotic transcriptome data analysis, the expressions of *MsYUC1* and *MsYUC10* were significantly increased under salt stress (**Figure 5C**) and the expressions of *MsYUC10* and *MsYUC12* were induced by cold (**Figure 5D**). Mannitol treatment significantly induced the expression of *MsYUC10* (**Figure 5E**). RT-qPCR analysis further confirmed that *MsYUC1* and *MsYUC10* expression could be induced by NaCl (100 mM) and *MsYUC10* and *MsYUC12* could be elevated by cold (4°C) for 3 h treatments (**Figures 5F,G**). Expression analysis showed that *MsYUC* genes might have a tissue-specific expression and differential abiotic stress response pattern.

### 3.7 Prediction of the protein interaction network and targeted miRNA of MsYUC members

**Figure 6:**
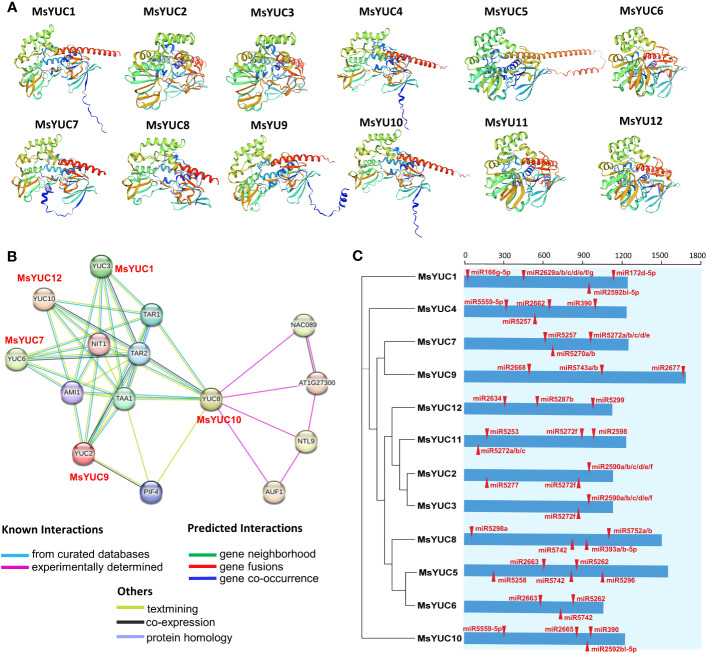
Predicted protein interaction network of MsYUC proteins and miRNA target sites in *MsYUC* genes. **(A)** Protein structure prediction of MsYUCs. **(B)** Protein interaction network predicted using MsYUC orthologs from *Arabidopsis*. **(C)** Predicted miRNA targets in the *MsYUC* coding sequence. The red tangles represent the miRNA-targeted *MsYUC* sites.

Protein structure prediction showed that MsYUC proteins shared a unique topology containing several α-helices and β-structures (**Figures 6A**, **S2**), indicating structural conservation. The trans-membrane prediction showed that MsYUC4, 9, 10, and 11 proteins possessed one TMD, respectively. The TMD regions of MsYUC9 and 11 proteins were localized in the N-terminal, whereas the TMD regions of MsYUC4 and 10 were localized in the middle of the protein (**Figure S3**). A predicted protein interaction network indicated that MsYUC proteins had multiple interaction partners (**Figure 6B**). MsYUC10 protein was predicted to interact with transcription factor NAC089 and NAC-like NTL9, and auxin upregulated F-box protein 1 (AUF1), which is a component of E3 ubiquitin ligase complexes. Both MsYUC9 and MsYUC10 proteins were predicted to interact with phytochrome interacting factor 4 (PIF4). MsYUC9 protein could also interact with TAA1 and amidase 1 (AMI1), which functions in auxin biosynthesis. MsYUC1, 7, 9, 10, 12 were predicted to interact with TAA1, TAR1, and TAR2, which function in the first step of the IPA pathway. We next performed miRNA target site prediction for the *MsYUC* genes. As shown in **Figure 6C**, *MsYUC2, 3*, and *11* were predicted to be targeted by a similar miRNA5272f. *MsYUC5, 6*, and *8* were predicted to be targeted by a similar miRNA5742. All the coding sequences of *MsYUCs* contained at least three predicted targets for miRNA.

## 4 Discussion

The YUC gene family proteins involved in auxin biosynthesis are the first identified FMO class family in plants that regulate growth, development, and tolerance in plants (Zhao et al., 2001). In *Medicago*, the YUC number (12 *MsYUCs* and 15 *MtYUCs*) was close to 11 *AtYUC* in *Arabidopsis* and 14 *OsYUC* in rice (Yamamoto et al., 2007; Zhao, 2012). Gene duplication is thought to be the main driver of species evolution and a direct cause of gene family expansion (Lynch and Conery, 2000; Moore and Purugganan, 2003; Maere et al., 2005), and two forms of gene duplication (tandem and segmental) events were identified in *Medicago* YUC gene families. In the MtYUC gene family, there was a gene cluster containing three *MtYUC* members (*MtYUC5/6/7*). Moreover, there was no distribution of *MsYUC* genes on chromosomes 2, 4, and 8 (**Table 1**, **Figure S1A**) whereas *MtYUC* genes were distributed in all chromosomes except for chromosome 2 (**Table 1**; **Figure S1B**). These reasons may together contribute to the more members of MtYUC than that of MsYUC. Notably, most of the YUC proteins in rice (Monocots) and *Medicago* or *Arabidopsis* (Dicots) could not gather under the same branch, as clade II-4 had no rice YUC protein and clade I-3 and clade II-3 only contained rice YUC proteins, indicating that YUC proteins underwent an evolutionary divergence, as that there are missing or duplication of YUCs during evolution. (**Figure 1**). In addition, Ka/Ks was used to evaluate their specific positions under positive selection pressure after duplication (Lynch and Conery, 2000; Mayrose et al., 2007). In this study, the Ka/Ks value of each duplication gene pair of YUCs of *Medicago* for all gene pairs was less than 1 (**Table S3**), which suggested that these genes had evolved under strong purifying selection. Since the divergence time of the Papilionoideae subfamily, which includes the genus *Medicago*, was approximately 34–63.7 millions of years (MYA) (Wang et al., 2023), the evolutionary divergence time of homologous gene pairs *MsYUC4/10* and *MtYUC1/8* was derived from the formation period of Papilionoideae subfamily. Because of the importance of *M. sativa* with high yield, nutrient value, and palatability, the mechanisms regulating its growth are of significant interest (Yamamoto et al., 2007). Functional orthologs of *YUC* genes in model species can provide insight into the functions in *Medicago* (Wei and Gai, 2008). MtYUC2 showed a close relationship with AtYUC1 and AtYUC4 (**Figure 2**), which have been reported to play vital roles in the formation of floral organs and vascular tissues in *Arabidopsis* (Cheng et al., 2006). However, the *Arabidopsis* AtYUC1 and AtYUC4 had no corresponding homologs in the *M. sativa* genome. Moreover, some YUCs of *M. sativa* had no homologs in *Arabidopsis* or rice, indicating that the gene loss event may have occurred after species divergence (**Figure 1**; **Table S2**).

In *Arabidopsis*, the roots and shoots appear to use two separate sets of *YUC* genes for auxin biosynthesis: ER-located YUCs functioning in roots or cytoplasmic-located YUCs functioning in shoots (Kriechbaumer et al., 2015). Phylogenetic tree analysis showed that *AtYUC3, 5, 7, 8*, and *9*, which were reported to function in roots with ER location, clustered in clade I-1 (Kriechbaumer et al., 2015). *MsYUC4* and *MsYUC10*, closely related to *AtYUC5, 8, 9*, also showed a predicted cytoplasmic location (**Table 1**). In *M. sativa*, *MsYUCs* also showed different expression patterns in different tissues. For example, *MsYUC10*, *MsYUC12*, and *MsYUC2* had relatively higher expression levels in specific tissues examined and *MsYUC2* had a higher expression level in leaves than in other tissues (**Figure 4A**). *MsYUC9*, closely related to *AtYUC2*, which was reported to function in shoots, was expressed not only in the shoots but also in roots of *M. sativa*. *MsYUC12*, with no homologous genes in *Arabidopsis*, showed higher expression levels in all tissues and was inferred to have universal roles during plant growth and development (**Figure 5A**). Therefore, in contrast to *AtYUC* expression, *MsYUC* expression does not seem to be clearly divided into shoot or root independent expression, suggesting a specificity in *M. sativa* compared with *Arabidopsis*.

The IPA-dependent pathway also plays an important role in integrating environmental stress and hormone signaling, and YUCs were reported to be involved in environmental stress response (Blakeslee et al., 2019). Cis-acting elements on the *MsYUCs*’ promoter revealed a variety of stress response elements related to hormone such as Auxin-, ABA-, JA-, GA-, and SA-responsive elements in the promoters of certain *MsYUC* genes (**Figure 5A**). In *Arabidopsis*, ABA can inhibit the transcription of *YUC2/8* via ABI4, thereby inhibiting primary root elongation (Yu et al., 2014). JA has been reported to promote lateral root growth through a direct regulation of *YUC2* by transcription factor ERF109 (Cai et al., 2014). JA also directly activates YUC8/9-dependent auxin biosynthesis to function in mechanical wounding response (Perez-Alonso et al., 2021). In *M. sativa*, six ABRE elements were found in the promoter of *MsYUC8* and five JA response elements were found in the promoter of *MsYUC7*, respectively. In addition, MsYUC7 showed a closer relationship with AtYUC2, implying a similar function in JA response. In *Arabidopsis*, expression levels of *YUC7, 9, 10*, and *11* were upregulated under dehydration conditions (Shi et al., 2014). Activation of *YUC7* enhances drought resistance in *Arabidopsis* (Lee et al., 2012). Overexpressed *YUC6* of *Arabidopsis* in potato and poplar plants or overexpressed *BnaYUC6a* in *Arabidopsis* and oilseed rape showed typical auxin overproduction alternation and conferred high drought resistance (Kim et al., 2012; Ke et al., 2015; Hao et al., 2022). Since *MsYUC11*, which is closely related to *AtYUC6*, had a five-element response to osmotic stress but no drought response elements, suggesting a function differentiation among species (**Figure 5A**).

*YUC* expression was also reported to be affected by cold stress. For example, cucumber *CsYUC10b* was upregulated by cold stress whereas other *CsYUCs* were downregulated (Yan et al., 2016). In the hypocotyl, the PIF4-YUC8 regulatory module plays an important role in response to stress signals, including light stress. The accumulation and transcriptional activity of PIF4 are regulated by different proteins, with competition for and interference at the *AtYUC8* promoter by other transcription factors affecting the positive regulation of *AtYUC8* by PIF4 and consequently affecting biosynthesis of auxin (Ma et al., 2016). In this study, all the *MsYUC* promoters had light-response elements. *MsYUC1, 2*, and *8* promoters had the most light-response elements, whereas the *MsYUC12* promoter had the least light-response element (**Figure 5A**). MsYUC10 was further predicted to interact with PIF4, indicating a similar function with AtYUC8 in light response (**Figure 6B**). Transcription factor AGL21 positively regulates *AtYUC5/8* which could be induced by IAA/ABA/JA and a variety of stresses, including salt stress (Yu et al., 2014). MsYUC1 and MsYUC10, which were clustered in the same sub-clade with AtYUC3/5/7/8/9, showed a significantly salt-induced expression (I-1), indicating a salt-response function in *M. sativa* (**Figure 5F**). The promoter of *MsYUC1, 2*, and *3* contained one cis-acting element (LTR) involved in low-temperature responsiveness, respectively (**Figure 5A**). Cold stress significantly elevated the expression of *MsYUC10* and *MsYUC12*, indicating an LTR-independent cold stress response function (**Figure 6B**). Moreover, *MsYUC10* and *MsYUC12* showed a higher expression level in 95-608 compared with other genotypes. Therefore, the stress tolerance of 95-608 should be further compared with other varieties. Studies indicate that miRNA-directed regulation of transcription factors may also play key roles in the precise regulation of IPA-dependent auxin biosynthesis in plants (Luo and Di, 2023). In this study, all *MsYUCs* contained at least three predicted targets for miRNA, suggesting a miRNA-directed regulation of *YUC* in *M. sativa* (**Figure 6C**).

## 5 Conclusion

In this study, the YUCs of *M. sativa* and *M. truncatula* were identified on a genome-wide scale. The phylogenetic analysis and comparative syntenic maps of *M. sativa* with other species illustrated their evolution relationship. The tissue and genotype-specific expression and abiotic stress response profiles have also been analyzed to reveal potential functional *YUC* genes. Moreover, RT-qPCR verified that certain *MsYUC* members represented salt or cold stress-affected expression patterns. Results in this study could provide valuable information for functional analysis and for the underlying regulation mechanism study of a specific *MsYUC* gene of *M. sativa*, especially under different tissues and various abiotic stresses through modification of the auxin synthetic IPA pathway in *Medicago*.

## Data availability statement

The original contributions presented in the study are included in the article/**Supplementary Material**. Further inquiries can be directed to the corresponding authors.

## Author contributions

AS: Conceptualization, Data curation, Methodology, Writing – original draft. SF: Methodology, Software, Writing – original draft. XX: Validation, Investigation, Writing – review & editing. WW: Funding acquisition, Resources, Writing – review & editing. JF: Project administration, Supervision, Writing – review & editing.
