# Supplementary material for: Identification and evolution analysis of YUCCA genes of Medicago sativa and Medicago truncatula and their expression profiles under abiotic stress
Source: Front Plant Sci. 2023 Aug 28;14:1268027. doi: 10.3389/fpls.2023.1268027 (PMC10494245; doi:10.3389/fpls.2023.1268027)
Supplement: Supplementary file 1 [file DataSheet_1.docx]

Supplementary Material

Identification and evolution analysis of flavin-containing monooxidases (YUCCA) genes of *Medicago* and their expression profiles under abiotic stress

An Shao, Shugao Fan, Xiao Xu, Wei Wang*, Jinmin Fu*

*** Correspondence:** W.W: weiwang@ldu.edu.cn; J.F: turfcn@qq.com


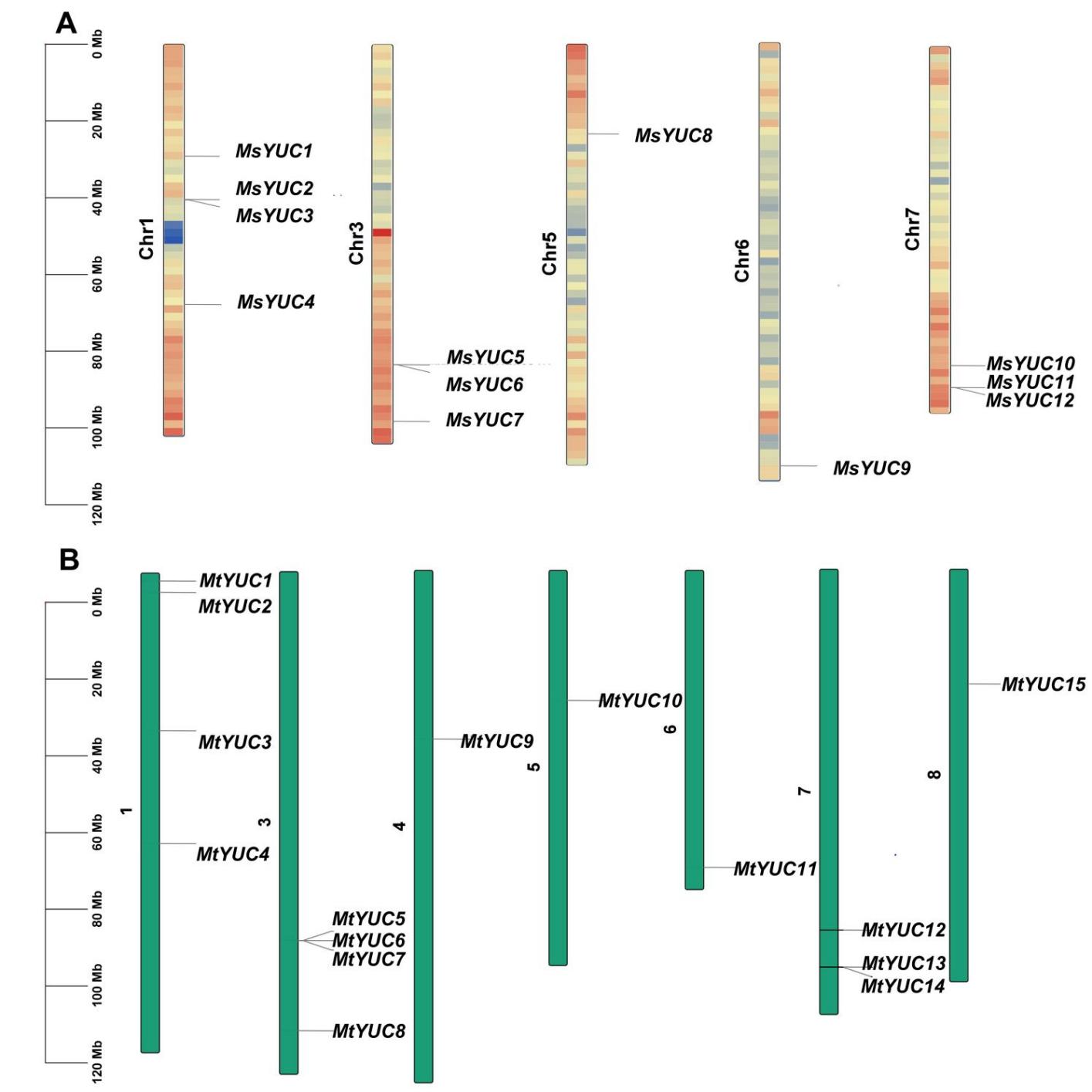


**Supplementary Figure 1.** Chromosomal mapping of the YUC gene family in *Medicago.* Distribution of *MsYUCs* genes in alfalfa Chromosome **(A)** and *MtYUCs* genes in **(B)** *M. truncatula* chromosome.

*
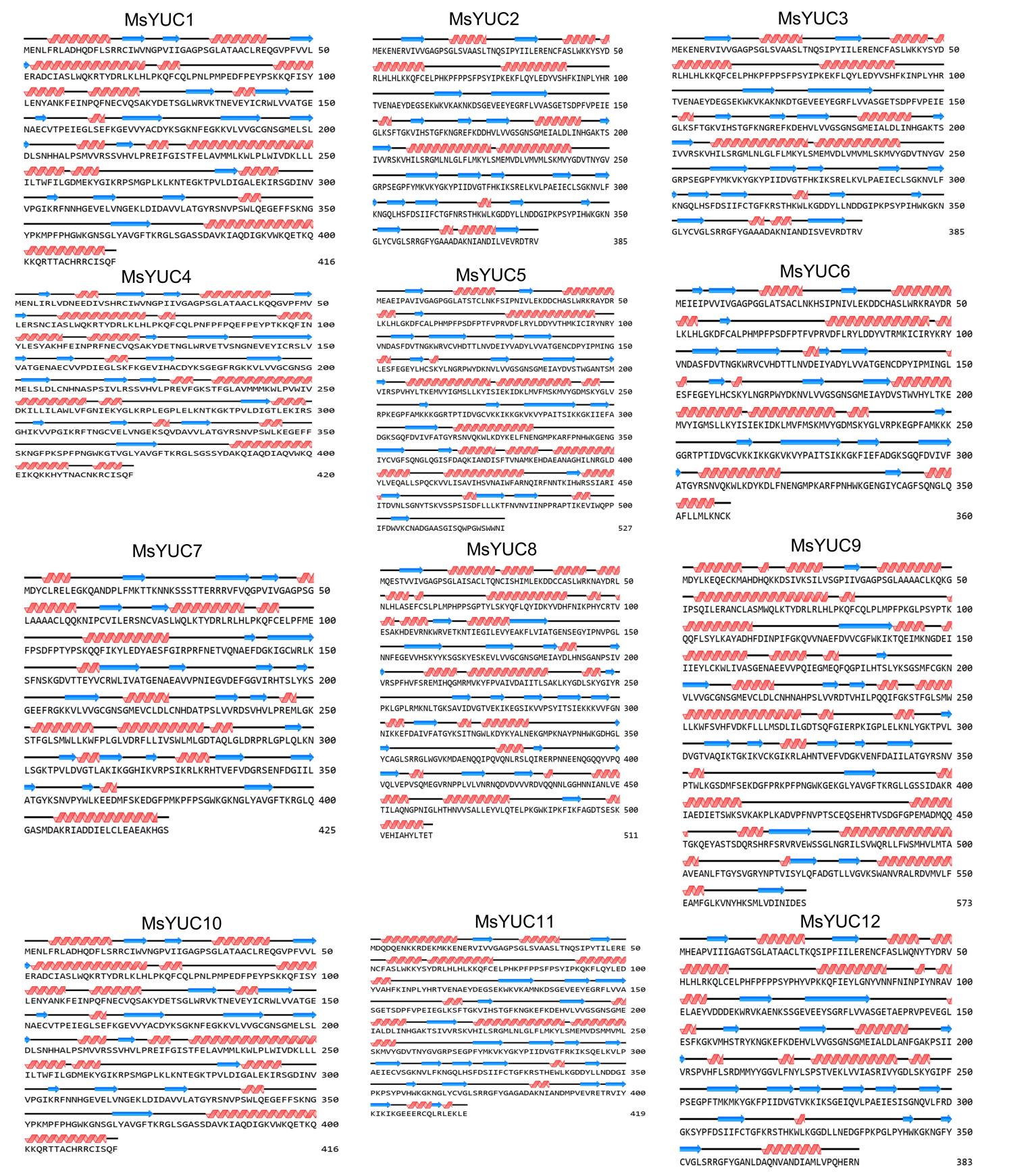
*

**Supplementary Figure 2.** Secondary structure prediction of MsYUCs by Phyre2. α-helices and β-structures were presented by red helics and blue arrow, respectively. http://www.sbg.bio.ic.ac.uk/ servers/phyre2/html/page.cgi? id=index

**
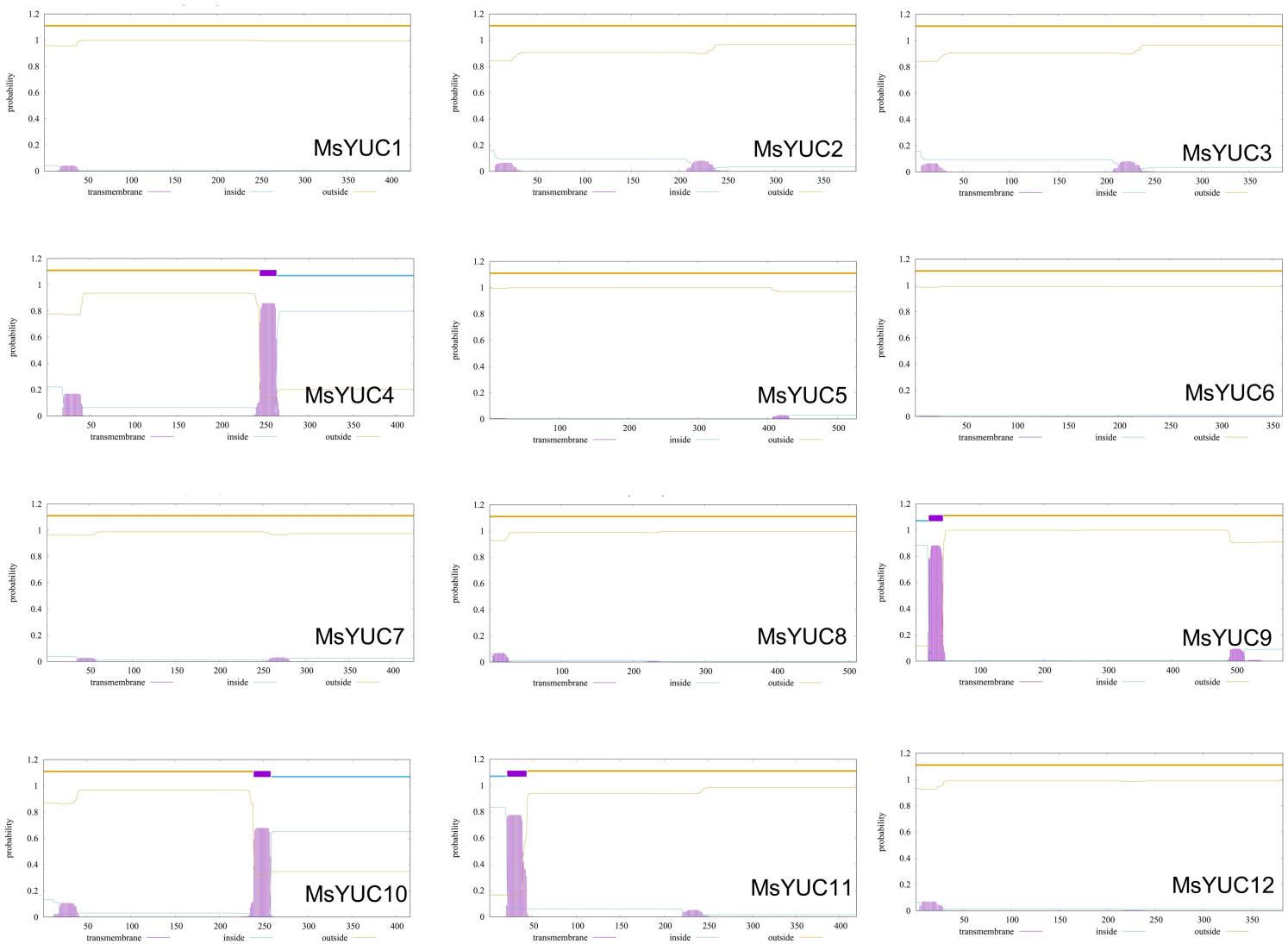
**

**Supplementary Figure 3.** Trans-membrane domains (TMD) prediction of MsYUCs. https://services.healthtech.dtu.dk/services/TMHMM-2.0/. The potential TMD was indicated as purple color.

**Supplementary Table 1.** Primers used for RT-qPCR.

| **Gene** | **Forward primer sequence 5’-3’** | **Reverse primer sequence 5’-3’** |
| --- | --- | --- |
| *MsYUC1* | GGAGCTGGTCCATCAGGC | GCCAAAGGGAAGCAATGCA |
| *MsYUC2* | GAGAGTTTAAAGATGATCATG | GTTTTAGCACCATGGTTGAT |
| *MsYUC3* | TCCCAAGCCAAGTTACCCT | GCATCTGCAGCAGCTCCA |
| *MsYUC4* | ACCCACGATTCAACGAGTGT | AGCAACCACAAGCGACCT |
| *MsYUC5* | CCTCCTAGGGCCCCTACC | CCCAGAAGCAGCCCCATC |
| *MsYUC6* | TTGTAGGTGCTGGGCCTG | CCTCCAGAGAGATGCATGGC |
| *MsYUC7* | TCGGGTTGTCCATGTGGT | GGGACCCAAACGAGGACG |
| *MsYUC8* | ATGCCTCACCCACCCTCA | AGCAGACTCAACTGTGCGA |
| *MsYUC9* | GTTTCGGACGGGTTCGGT | ACCGCTGCTCCACTCAAC |
| *MsYUC10* | TTCTCTCACGCCGTTGCA | AAGACATGCTGCCGTTGC |
| *MsYUC11* | CCAAGCCAAGTTACCCCGT | CATCCGCACCAGCTCCAT |
| *MsYUC12* | TCTGTCACCAAGCACGGT | TGGTGAACGGGCCTTCAC |

**Supplementary Table 2.** Collinearity analysis of *M. sativa* with *M. truncatula* or *A. thaliana* or rice.

| **Ms** | | | **At** | | |
| --- | --- | --- | --- | --- | --- |
| Chr1 | MsG0180003762.01.T01 | *MsYUC4* | Chr1 | transcript:AT1G04180.1 | *AtYUC9* |
| Chr1 | MsG0180003762.01.T01 | *MsYUC4* | Chr1 | transcript:AT1G04610.1 | *AtYUC3* |
| Chr1 | MsG0180003762.01.T01 | *MsYUC4* | Chr4 | transcript:AT4G28720.1 | *AtYUC8* |
| Chr1 | MsG0180003762.01.T01 | *MsYUC4* | Chr5 | transcript:AT5G43890.1 | *AtYUC5* |
| Chr3 | MsG0380016438.01.T01 | *MsYUC5* | Chr1 | transcript:AT1G21430.1 | *AtYUC11* |
| Chr3 | MsG0380017591.01.T01 | *MsYUC7* | Chr5 | transcript:AT5G25620.2 | *AtYUC6* |
| Chr6 | MsG0680035661.01.T01 | *MsYUC9* | Chr4 | transcript:AT4G13260.1 | *AtYUC2* |
| Chr7 | MsG0780040831.01.T01 | *MsYUC10* | Chr1 | transcript:AT1G04180.1 | *AtYUC9* |
| Chr7 | MsG0780040831.01.T01 | *MsYUC10* | Chr4 | transcript:AT4G28720.1 | *AtYUC8* |
| Chr7 | MsG0780040831.01.T01 | *MsYUC10* | Chr5 | transcript:AT5G43890.1 | *AtYUC5* |
| **Ms** | | | **Mt** | | |
| Chr1 | MsG0180003762.01.T01 | *MsYUC4* | Chr1 | KEH42432 | *MtYUC4* |
| Chr1 | MsG0180001906.01.T01 | *MsYUC1* | Chr1 | KEH41176 | *MtYUC3* |
| Chr1 | MsG0180003762.01.T01 | *MsYUC4* | Chr7 | AES81674 | *MtYUC12* |
| Chr3 | MsG0380017591.01.T01 | *MsYUC7* | Chr1 | AES58795 | *MtYUC1* |
| Chr3 | MsG0380016438.01.T01 | *MsYUC5* | Chr3 | KEH35392 | *MtYUC5* |
| Chr3 | MsG0380017591.01.T01 | *MsYUC7* | Chr3 | AES73853 | *MtYUC8* |
| Chr6 | MsG0680035661.01.T01 | *MsYUC9* | Chr6 | KEH27129 | *MtYUC11* |
| Chr7 | MsG0780040831.01.T01 | *MsYUC10* | Chr1 | KEH42432 | *MtYUC4* |
| Chr7 | MsG0780040831.01.T01 | *MsYUC10* | Chr7 | AES81674 | *MtYUC12* |
| Chr7 | MsG0780041255.01.T01 | *MsYUC11* | Chr7 | KEH24362 | *MtYUC13* |
| **Ms** | | | **Os** | | |
| Chr6 | MsG0680035661.01.T01 | *MsYUC9* | Chr1 | transcript:Os01t0224700-01 | *OsYUC4* |

**Supplementary Table 3.** The Ka/Ks ratios and the divergence time for orthologous YUC genes of *Medicago*.

| **Gene ID** | **Gene ID** | **Ka** | **Ks** | **Ka/Ks** | **MYA** |
| --- | --- | --- | --- | --- | --- |
| MsYUC4 | MsYUC10 | 0.12004258 | 0.62190101 | 0.19302522 | 47.838539 |
| MtYUC1 | MtYUC8 | 0.15233446 | 0.74470308 | 0.20455731 | 57.284852 |
| MsYUC5 | MsYUC6 | 0.03262154 | 0.06618464 | 0.49288687 | 5.0911268 |
| MsYUC11 | MsYUC12 | 0.22755607 | 0.98509682 | 0.23099868 | 75.776678 |
| MtYUC5 | MtYUC6 | 0.00548799 | 0.06037452 | 0.09089921 | 4.6441938 |
| MtYUC6 | MtYUC7 | 0.01657984 | 0.06889755 | 0.24064485 | 5.2998115 |
| MsYUC4 | MtYUC4 | 0.01138541 | 0.06561559 | 0.17351696 | 5.0473531 |
| MsYUC1 | MtYUC3 | 0.00925303 | 0.13553662 | 0.06826959 | 10.425894 |
| MsYUC4 | MtYUC12 | 0.12275669 | 0.63517838 | 0.19326334 | 48.859875 |
| MsYUC7 | MtYUC1 | 0.14571885 | 0.80250514 | 0.18157995 | 61.731165 |
| MsYUC5 | MtYUC5 | 0.03359284 | 0.07398601 | 0.45404311 | 5.6912319 |
| MsYUC7 | MtYUC8 | 0.01394188 | 0.07759317 | 0.17967922 | 5.9687057 |
| MsYUC9 | MtYUC11 | 0.01185170 | 0.04929309 | 0.24043342 | 3.7917768 |
| MsYUC10 | MtYUC4 | 0.11823912 | 0.61728125 | 0.19154821 | 47.483173 |
| MsYUC10 | MtYUC12 | 0.00519392 | 0.05899846 | 0.08803494 | 4.5383434 |
| MsYUC11 | MtYUC13 | 0.03829560 | 0.15401735 | 0.24864471 | 11.847488 |

**Supplementary Table 4.** Conversed motif of YUC gene family members in Medicago.

| **ID** | MsYUC |  | MtYUC |
| --- | --- | --- | --- |
| Motif1 | GAGPSGLATAACLTQQSIPYIILEREBCFASLWQKRTYDRLKLHLPKQFC | Motif1 | GAGPSGLATAACLKQQSIPFIILERDBCIASLWQKRTYDRLKLHLPKQFC |
| Motif2 | PTYPPKKQFJQYLEDYVKHFKINPRYNRTVZNAEYDETSGKWRVKTKNT | Motif2 | YKSGREFKDKNVLVVGCGNSGMEIALDLCNHGANPSIVVRSPVHVLPREM |
| Motif3 | DGFPKPPFPNHWKGKNGLYCVGFSRRGLYGASADAKNIAND | Motif3 | ELPHMPFPSDFPTYPPKVQFJRYLEDYVKHFKINPRYNRTVESAEFDET |
| Motif4 | AEPVVPEIEGLESFEGEVIHSTDYKNGREFKDKNVLVVGSGNSGMEIALD | Motif4 | MPKKPFPBGWKGENGLYAVGFTKRGLQGASFDAKKIANDIAFTWKAEKKH |
| Motif5 | YGDVSKYGLKRPSEGPLEMKNKYGKTPVJDVGTVEKIKSGEIKVLPAIKR | Motif5 | SKLVYGDMSKYGJKRPKEGPLELKNKTGKTPVJDVGTVKKIKSGKIKVVP |
| Motif6 | SGKNVEFVBGKSHSFDSIIFATGYRSNVPKWLKEDDYLSN | Motif6 | GKIVEFVDGKSGQFDAIVFATGYRSNVPKWLKD |
| Motif7 | LCNHGAKPSIVVRSSVHILPREMLGKGTF | Motif7 | YVARWLVVATGENAEPVVPEIEGLESFEG |
| Motif8 | EEYEGRFLVVATGEN | Motif8 | JGMSLLKYLPVEKVDKLLLLL |
| Motif9 | LLKYLSIEMVDKJLVILSKMV | Motif9 | LNDKGMPKNAYPNHWKGDNGLYCAGLARR |
| Motif10 | ZLPHMPFPPSF | Motif10 | KWRVCVKBTTLNIDE |
| Motif11 | WKQETKQKKQRTTACHRRCIS | Motif11 | RCIWVNGPIIV |
| Motif12 | DPEDILSHRCIWVNGPVI | Motif12 | KQRTTACHRRCISQF |
